# On Some Properties of Whyburn Spaces

**DOI:** 10.1155/2022/7113360

**Published:** 2022-02-09

**Authors:** Abdelwaheb Mhemdi, Sami Lazaar, Khedidja Dourari

**Affiliations:** ^1^Prince Sattam Bin Abdulaziz University, College of Sciences and Humanities in Aflaj, Al-Kharj, Saudi Arabia; ^2^Taibah University, Medina, Saudi Arabia; ^3^Tunis El Manar University, Tunis, Tunisia

## 1. Introduction

Alexandroff space is a topological space such that its collection of open sets is closed under arbitrary intersection. In 1937, Alexandroff introduced these spaces with the name of “Diskrete Räume” [1]. In [2], Steiner has named them principal spaces. Alexandroff spaces are used and applied in different domains like geometry, theoretical physics, and diverse branches of computer sciences. After that, Alexandroff spaces played an important role in digital topology (cofinite spaces) (see [3–6]).

The specialization quasiorder of an Alexandroff space is defined by

(1) $$\begin{array}{c} x\leq y⟺x\in \bar{\left\{y\right\}}. \end{array}$$

Now, if ≤ is a quasiorder on space *X* then the set of all supersets ℬ:={*x*↑:*x* ∈ *X*} (*x*↑:={*y* ∈ *X* : *x* ≤ *y*}.) forms a basis of an Alexandroff topology Y(≤) on *X*. In this case, the closure $\bar{\left\{x\right\}}$ is exactly the downset *x* ↓ :={*y* ∈ *X* : *y* ≤ *x*}. We denote by *v*(*x*)=*x*↑ the minimal neighborhood of *x*. For more information on Alexandroff spaces you can see [7–13].

In [14], Echi introduced a particular class of Alexandroff spaces named primal spaces. (*X*, *τ*) is called a primal space if there exists a map *f* : *X*⟶*X* such that *τ*=Y(*f*), where Y(*f*) is the collection of all *f*-invariant subsets of *X* (for more information see [14, 15]). In [16], the authors characterized maps *f* such that the primal space (*X*, Y(*f*)) is submaximal or door.

This paper is devoted to characterizing Alexandroff spaces which are submaximal, door, *n*-resolvable, Whyburn, and weakly Whyburn. Some useful examples are presented and commented and finally, all results on primal spaces are deduced. In the first section of this paper, we will give characterizations of Alexandroff spaces to be a submaximal, door, and *n*-resolvable. The second section is devoted to introducing and characterizing topological spaces, called quasi-Whyburn spaces, such that their *T*_0_-refections are Whyburn. Particularly, the case of Alexandroff spaces is totally deduced in this particular class of spaces.

## 2. Submaximal, Door, and *n*-Resolvable Alexandroff Spaces

We know that a submaximal space is a topological space in which all dense subsets are open.

The following theorem characterizes submaximal spaces [17].

**Theorem 1 .**

A topological space *X* is submaximal if and only if for every *Y*⊆*X*, the subset $\bar{Y}\setminus Y$ is closed equivalently for every *Y*⊆*X* and the subset $\bar{Y}\setminus Y$ is closed and discrete.

Now, before giving the first main result of this section, some useful examples are presented and commented.

**Example 1 .**

1. Let *a*, *b*, *c*, and *d* be distinct points and *X*={*a*, *b*, *c*, *d*} equipped with the topology *τ*={∅, *X*, {*a*, *b*, *c*}, {*b*}, {*c*}, {*b*, *c*, *d*}, {*b*, *c*}}. We can find the specialization quasiorder as follows: $\bar{\left\{a\right\}}=\left\{a\right\}$, $\bar{\left\{d\right\}}=\left\{d\right\}$, $\bar{\left\{b\right\}}=\left\{a,b,d\right\}$, and $\bar{\left\{c\right\}}=\left\{a,c,d\right\}$ so that *a* ≤ *b*, *d* ≤ *b*, *a* ≤ *c*, and *d* ≤ *c*.
2. We have (*X*, *τ*) is an Alexandroff space which is submaximal.
3. Consider the set *ℕ* of natural numbers and for each *n* ∈ *ℕ*, let *𝒪*_*n*_={0,1,2,…, *n*}. We equip the space *ℕ* with the topology *τ*={∅, *ℕ*, {1}} ∪ {*𝒪*_*n*_, *n* ∈ *ℕ*}.

The Alexandroff space *ℕ*, *τ* is not submaximal. In fact, *ℕ*∖{2} is a dense subset which is not open.

The previous examples give a motivation to investigate conditions allowing Alexandroff spaces to be submaximal.

The answer is given by the following theorem.

**Theorem 2 .**

Let *X* be an Alexandroff space. Then, the following statements are equivalent:

1. *X* is submaximal
2. The specialization quasiorder is an order and every chain in its graph has a length less than or equal to 2

**Proof**

(i) [(1)⟹(2)]. Suppose that *X* is submaximal. If *x*, *y* are two elements of *X* satisfying *x* ≤ *y* and *y* ≤ *x* then $x\in \bar{y}$ and $y\in \bar{x}$, which imply that $\bar{\left\{x\right\}}=\bar{\left\{y\right\}}$. Using the fact that every submaximal space is *T*_0_, we deduce that *x*=*y* and then the specialization quasiorder is an order.
Now, suppose that *x* < *y* < *z*. If *Y*={*x*, *z*}, then $y\in \bar{Y}\setminus Y$ and $x\in \bar{y}\subseteq \bar{\bar{Y}\setminus Y}$. Since $x\notin \bar{Y}\setminus Y$ then it is not closed.
(ii) [(2)⟹(1)]. If *Y* is a subset of *X*, we denote $Y_{c}=\left\{a\in Y:\bar{\left\{a\right\}}\neq \left\{a\right\}\right\}$. Using 2), for every *a* ∈ *Y*∖*Y*_*c*_ and for any $b\in \bar{\left\{a\right\}}\setminus \left\{a\right\}$, {*b*} is closed. Since *X* is an Alexandroff space, then
  (2) $$\begin{array}{c} \bar{Y}=\underset{a\in Y}{\cup }\bar{\left\{a\right\}}\\ =A\cup \left(\underset{a\in Y-Y_{c}}{\cup }\bar{\left\{a\right\}}\setminus \left\{a\right\}\right), \end{array}$$
so that $\bar{Y}\setminus Y$ is closed and then *X* is submaximal.

**Corollary 1 .**

(see [14], Theorem 4.1)

If (*X*, *f*) is a flow in set, then (*X*, Y(*f*)) is a submaximal space if and only if *f*^2^=*f*.

**Definition 1 .**

Let *X* be a topological space. *X* is called a door space if any subset of *X* is open or closed.

Now, we state straightforward remarks.

**Remark 1 .**

1. The example cited in Example 1 (1)provides a space that is not a door. Indeed, the subset {*a*, *c*} is neither open nor closed.
2. Let *X* be the set {(1/*n*), *nℕ*^*∗*^} ∪ {0} equipped with the topology defined as follows: for each *x* ∈ *X*∖{0}, *v*(*x*)={*x*} and *v*(0)=*X*.

Hence, every subset of *X* not containing 0 is open. Yet, every subset of *X* containing 0 is closed.

Therefore, *X* is an Alexandroff door space.

Considering Alexandroff door spaces, the second main result of this section is given by the following theorem. But, first, we need to recall increasing and decreasing sets.

The increasing hull of a set *A* in a quasiordered set (*X*, ≤) is *i*(*A*)={*x* ∈ *X* : *x* ≥ *a* for some *a* ∈ *A*}. A set *A* is increasing if *A*=*i*(*A*). The set *i*({*x*}) may be written as *i*(*x*). Decreasing hulls and decreasing sets are defined dually. The closed sets in an Alexandroff space are just the decreasing sets for the specialization quasiorder, and the open sets are just the increasing sets.

**Theorem 3 .**

Let *X* be an Alexandroff space and ≤ is its specialization order. Then, the following statements are equivalent:

1. *X* is a door space
2. The length of every chain in the graph of ≤ is not greater than 2 and all chains of length 2 contain a common point which must be a maximal point *M* or a minimal point *m*

**Proof**

- [(2)⟹(1)]. Suppose that the common point is a minimal point *m*. Let *Y* be a nonclosed subset of *X*. So that *Y* is not decreasing in (*X*, ≤) equivalently there is *x* ∈ *Y* such that *x* ∈ *i*(*m*) and *m* ∉ *A*. Since a subset which not contains *m* is increasing, therefore, it is open.
- We work dually if the common point is a maximal point.
- [(1)⟹(2)]. By contradiction suppose that either there exist *a* < *b* < *d* or *a* < *b* and *c* < *d* of length 2 with no common point. In these cases, we have always {*a*, *d*} is not increasing and not decreasing, thus it is not open and not closed which is a contradiction. This fact completes the proof.

**Corollary 2 .**

(see [16], Theorem 4.3)

If (*X*, *f*) is a flow in set, then (*X*, Y(*f*)) is a door space if and only if |*f*(Fix(*f*)^*c*^)| ≤ 1.

Now, we will give a study of *n* − resolvable Alexandroff spaces.

First, let us recall the definition of *n* − resolvable spaces. If *X* is a topological space, then it is called *n* − resolvable (*n* > 1) if there exist *n*-many mutually disjoint dense sets of *X*. A 2-resolvable space is called a resolvable space. Hewitt added also the condition “has no isolated points” to the definition of resolvable spaces. Also, a topological space is *n* − resolvable if and only if it is the union of *n*-many mutually disjoint dense subsets.

Stone [18] characterizes Alexandroff spaces which are *n*-resolvable in the following theorem.

**Theorem 4 .**

(see [18]). Let (*X*, ≤) be a quasiordered set. Then, we have equivalence between the following statements:

1. *X* admit a partition into *n* mutually disjoint cofinal sets
2. ∀*x* ∈ *X*, *x*↑ has at least *n* elements

We note that, for every subset *Y* of an Alexandroff space *X*, we have equivalence between the following items:

1. *Y* is dense in *X*
2. $X=\bar{Y}=↓Y$
3. ∀*x* ∈ *X*, ∃*a* ∈ *Y* such that *x* ≤ *a*
4. *Y* is cofinal in (*X*, ≤)

This allows us to rephrase Stone's result as follows.

**Theorem 5 .**

Let (*X*, *τ*) be an Alexandroff space and ≤ is its specialization quasiorder. Then, the following statements are equivalent:

1. “≤” is *n*-resolvable
2. ∀*x* ∈ *X*, *x*↑ contains at least *n* elements
3. There is no maximal element in (*X*, ≤)
4. (*X*, *τ*) has no isolated points

We recall that the *T*_0_-reflection of a topological space *X* is the quotient space denoted by **T**_0_(*X*):=*X*/∼ obtained from the equivalence relation defined on *X* by *x* ~ *y* if and only if $\bar{\left\{x\right\}}=\bar{\left\{y\right\}}$.

**Corollary 3 .**

Let *X* be an Alexandroff space. Then the following statements are equivalent:

1. *X* is *n*-resolvable
2. ∀*x* ∈ *X*, *x*↑ contains at least *n* distinct points
3. Every maximal element *x* in the *T*_0_-refection *T*_0_(*X*) arises from a cycle *x*=*x*_1_ < *x*_2_ < *x*_3_ < ⋯<*x*_*n*_ < *x*_*n*_+1=*x* containing at least *n* distinct points *x*_*i*_

Now, we shed some light on interesting examples.

**Example 2 .**

1. Consider the set *ℤ* of all integers with the usual order ≤. For any integer *n* ≥ 2 let *A*_*k*_={*j* ∈ *ℤ* : *j*=*k* mod *n*}. Then *A*_0_, *A*_1_,…, *A*_*n*−1_ are mutually disjoint dense sets of the Alexandroff space (*ℤ*, Y(≤)), showing that this space is *n*-resolvable for every *n* ≥ 2. Indeed, it is obvious that *n*↑ is infinite for each *n* ∈ *ℤ*.
2. Let ≤ be the inverse order of *ℕ* where *ℕ* is the set of all natural numbers. In the Alexandroff space (*ℕ*, Y(≤)), every set *A* of *X* is dense if and only if 0 ∈ *A*. Therefore, (*ℕ*, Y(≤)) is not a resolvable space. In fact, we note that |0↑|=1.

## 3. Alexandroff Spaces Which Are Whyburn and Quasi-Whyburn Spaces

Let *X* be a topological space and *F* be a subset of *X*. Then, *F* is called almost closed if and only if $\bar{F}\setminus F=\left\{x\right\}$ for some *x* ∈ *X*. We use the notation *F*⟶*x*.

The notion of Whyburn spaces was first introduced as accessibility spaces by G.T. Whyburn in his famous paper [19]. Hence, a Whyburn space is a topological space *X* satisfying

(3) $$\begin{array}{c} Y\subseteq X,\\ \bar{Y}\neq Y,\\ x\in \bar{Y}\setminus Y⟹\exists B\subseteq Y\text{ s.t. }\bar{B}\setminus Y=\left\{x\right\}. \end{array}$$

A topological space *X* is called weakly Whyburn [20] if

(4) $$\begin{array}{c} Y\subseteq X,\\ \bar{Y}\neq Y⟹\exists B\subseteq Y\text{ s.t. }\bar{B}\setminus Y\text{ is a singleton.} \end{array}$$

We denote the class of all Whyburn spaces (resp., weakly Whyburn spaces) by AP-spaces (resp., WAP-spaces) [21–23].

### 3.1. Quasi-Whyburn Spaces

A continuous map *q* from a topological space *X* to a topological space *Y* is said to be a quasihomeomorphism if *U* ↦ *q*^−1^(*U*) defines a bijection between the collection of all open sets of *Y* and the collection of all open sets of *X* [24].

We can see easily that the canonical surjection *μ*_*X*_ : *X*⟶**T**_0_(*X*) is a quasihomeomorphism. More precisely, *μ*_*X*_ is an onto quasihomeomorphism, and in this case, the following results are useful.

**Lemma 1 .**

(see [25]). Let *μ* : *X*⟶*Y* be continuous onto the map. Then, *μ* is a quasihomeomorphism if and only if *μ* is an open map and *q*^−1^(*q*(*A*))=*A* for every open subset *A* of *X*; equivalently, *μ* is a closed map and *q*^−1^(*q*(*A*))=*A* for every closed subset *A* of *X*.

**Lemma 2 .**

(see [16]). A quasihomeomorphism *μ* : *X*⟶*Y* is onto if and only if $\mu^{-1}\left(\bar{A}\right)=\bar{\mu^{-1}\left(A\right)}$ for every subset *A* of *Y*.

If *X* is a topological space, *x* ∈ *X*, and *Y*⊆*X*, we take the notations in [16]. In that paper, authors denote by *d*_0_(*x*) the subset $\left\{y\in X:\bar{\left\{x\right\}}=\bar{\left\{y\right\}}\right\}$ and by *d*_0_(*Y*) the union of *d*_0_(*a*) for all *a* ∈ *Y*.

Using these notations, we can find the following properties:

- *d*_0_(*Y*)=*μ*_*X*_^−1^(*μ*_*X*_(*Y*))
- *d*_0_(*d*_0_(*Y*))=*d*_0_(*Y*)
- $Y\subseteq d_{0}\left(Y\right)\subseteq \bar{Y}$ and $\bar{d_{0}\left(Y\right)}=\bar{Y}$
- If *Y* is open or closed, then *d*_0_(*Y*)=*Y*

Now, we introduce the notions of *d*_0_-closed subsets, in a given topological space, and quasi-Whyburn spaces as follows.

**Definition 2 .**

Let *A* be a subset of a topological space *X*. Then,

- *A* is called *d*_0_-closed if *d*_0_(*A*) is closed, that is, if $d_{0}\left(A\right)=\bar{A}$
- If the *T*_0_-reflection of *X* is a Whyburn space, *X* is called a quasi-Whyburn space or a *QAP*-space (or also a *T*_0_-Whyburn space)

The following theorem gives a characterization of quasi-Whyburn spaces.

**Theorem 6 .**

If *X* is a topological space, then we have equivalence between the following statements:

1. *X* is a *QAP*-space
2. For all non-*d*_0_-closed subsets *A* of *X* and for all $x\in \bar{A}\backslash d_{0}\left(A\right)$, there is a subset *B* of *X* such that *d*_0_(*B*)⊆*d*_0_(*A*) with $\bar{B}\backslash d_{0}\left(B\right)=d_{0}\left(x\right)$

**Proof**

(1) ⟹ (2). Let *A* be a non-*d*_0_-closed subset of *X* and $x\in \bar{A}\backslash d_{0}\left(A\right)$. Then, $\mu_{X}\left(x\right)\in \bar{\mu_{X}\left(A\right)}\backslash \mu_{X}\left(A\right)$. By hypothesis, there is *B*⊆*X* such that *μ*_*X*_(*B*)⊆*μ*_*X*_(*A*) (which is equivalent to *d*_0_(*B*)⊆*d*_0_(*A*)) satisfying *μ*_*X*_(*B*)⟶*μ*_*X*_(*x*). Now, applying *μ*_*X*_^−1^, we have

(5) $$\begin{array}{c} d_{0}\left(x\right)=\mu_{X}^{-1}\left(\bar{\mu_{X}\left(B\right)}\setminus \mu_{X}\left(B\right)\right)\\ =\mu_{X}^{-1}\left(\bar{\mu_{X}\left(B\right)}\right)\setminus \mu_{X}^{-1}\left(\mu_{X}\left(B\right)\right)\\ =\bar{\mu_{X}^{-1}\left(\mu_{X}\left(B\right)\right)}\setminus \mu_{X}^{-1}\left(\mu_{X}\left(B\right)\right)\\ =\bar{d_{0}\left(B\right)}\setminus d_{0}\left(B\right)=\bar{B}\setminus d_{0}\left(B\right). \end{array}$$

(2) ⟹ (1). Conversely, let *A*⊆*X* such that *μ*_*X*_(*A*) is not closed in *T*_0_(*X*) and consider a point *x* in *X* with $\mu_{X}\left(x\right)\in \bar{\mu_{X}\left(A\right)}\backslash \mu_{X}\left(A\right)$. Then, $x\in \bar{A}\backslash d_{0}\left(A\right)$ with *A* non-*d*_0_-closed. So, by hypothesis, there is a subset *B* of *X* such that *d*_0_(*B*)⊆*d*_0_(*A*) (which is equivalent to *μ*_*X*_(*B*)⊆*μ*_*X*_(*A*)) satisfying $\bar{B}\backslash d_{0}\left(B\right)=d_{0}\left(x\right)$. Thus, $\mu_{X}^{-1}\left(\left\{\mu_{X}\left(x\right)\right\}\right)=\bar{\mu_{X}^{-1}\left(\mu_{X}\left(B\right)\right)}\setminus \mu_{X}^{-1}\left(\mu_{X}\left(B\right)\right)=\mu_{X}^{-1}\left(\bar{\mu_{X}\left(B\right)}\setminus \mu_{X}\left(B\right)\right)$. Therefore, *μ*_*X*_(*B*)⟶*μ*_*X*_(*x*).

**Definition 3 .**

A topological space *X* is called quasiweakly Whyburn space (or *T*_0_-weakly Whyburn space) and denoted by *QWAP*-space if its *T*_0_-reflection is a weakly Whyburn space.

The proof of the following result is similar to that of Theorem 6.

**Theorem 7 .**

If *X* is a topological space, then we have equivalence between the following statements:

1. *X* is a *QWAP*-space
2. For all non-*d*_0_-closed subset *A* of *X*, there is a subset *B* of *X* with *d*_0_(*B*)⊆*d*_0_(*A*) and $\bar{B}\backslash d_{0}\left(A\right)=d_{0}\left(x\right)$, for some *x* ∈ *X*

### 3.2. Alexandroff Spaces Which Are Whyburn and Quasi-Whyburn Spaces

**Theorem 8 .**

If *X* is an Alexandroff space, then we have equivalence between the following statements:

- *X* is Whyburn
- ∀*x* ∈ *X*,  |↓*x*| ≤ 2

**Proof**

Suppose that *X* is Whyburn and there exists *x* ∈ *X* such that (↓*x*)∖{*x*} contains two distinct elements *y* and *z*. Since {*x*} is not closed and $y\in \bar{\left\{x\right\}}\setminus \left\{x\right\}$, there exists *B* ⊂ {*x*} such that $\bar{B}\setminus \left\{x\right\}=\left\{y\right\}$. Yet, in that case, *B*={*x*}, which leads to a contradiction because (↓*x*)∖{*x*} contains also *z*.

Conversely, suppose that each element of *X* has at most 2 predecessors.

Let *Y*⊆*X* such that $\bar{Y}\neq Y$. Using the fact that *X* is Alexandroff, we have

(6) $$\begin{array}{c} \bar{Y}=\cup \left[↓t:t\in Y\right]. \end{array}$$

Let $x\in \bar{Y}\setminus Y$ and *t* ∈ *Y* satisfying *x* ∈ ↓*t*. Since |↓*t*| ≤ 2, then ↓*t*={*t*, *x*}. If we take *B*={*t*}, we can see that *B* ⊂ *Y* and $\bar{B}\setminus Y=\left\{x\right\}$. We deduce that *X* is a Whyburn space. □

**Corollary 4 .**

Let (*X*, *P*(*f*)) be a functionally Alexandroff space. Then, we have equivalence between the following statements:

- (*X*, *P*(*f*)) is a Whyburn space
- ∀*x* ∈ *X*,  *f*^2^(*x*) ∈ {*x*, *f*(*x*)}

**Example 3 .**

1. Consider a given set *X*={*a*, *b*, *c*, *d*}.
2. Let *τ*={∅, *X*, {*a*, *b*, *c*}, {*b*}, {*c*}, {*b*, *d*, *c*}, {*b*, *c*}} be an Alexandroff topology on *X*.
3. Suppose that *X* is Whyburn. Since {*b*} is not closed, then there exists *B* ⊂ {*b*} such that $\bar{B}\setminus \left\{b\right\}=\left\{a\right\}$. This is an impossible fact because $\bar{B}\setminus \left\{b\right\}=\left\{a,d\right\}$. Therefore, this Alexandroff space is not Whyburn.
4. Let *X*=*ℤ* ∪ {*∞*}. The topology on *X* satisfying $\bar{\left\{n\right\}}=\left\{n,\infty \right\}$, for every *n* ∈ *ℤ* and $\bar{\left\{\infty \right\}}=\left\{\infty \right\}$ is an Alexandroff topology.

**Figure 1:**
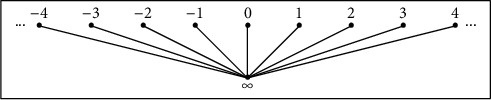
Alexandroff topology.

Clearly, for any nonempty subset *A* of *X*, we have $\bar{A}=A\cup \left\{\infty \right\}$; then, the condition $x\in \bar{A}\backslash A$ means that *x*=*∞*, and thus, *A*⟶*x*. We observe that, in this case, for every *n* ∈ *ℤ*, |↓*n*|=|{*n*, *∞*}|=2 and |↓*∞*|=|{*∞*}|=1. One can illustrate this situation in Figure 1.

**Remark 2 .**

1. Let (*X*, *τ*) be the Alexandroff space cited in Example 1 (1) and suppose that it is Whyburn. Since {*b*} is not closed, then there exists *B*⊆{*b*} such that $\bar{B}\setminus \left\{b\right\}=\left\{a\right\}$. This is an impossible fact because $\bar{B}\setminus \left\{b\right\}=\left\{a,d\right\}$. Therefore, this Alexandroff space is not Whyburn. Moreover, for the same reason, we note that the space cited in Example 1 (2) is not Whyburn.
2. The example cites in Remark 1 (2) provides a Whyburn space.

**Proposition 1 .**

Let *X* be an Alexandroff space. Then, *X* is weakly Whyburn if and only if *X* is Whyburn.

**Proof**

Clearly, any Whyburn space is weakly Whyburn.

Conversely, suppose that *X* is a WAP-space and there exists *x* ∈ *X* such that |↓*x*| > 2.

If we take *Y*={*x*}, then *Y* is not closed, which implies that there exists *B* ⊂ *Y* such that $\left|\bar{B}\setminus Y\right|=1$. Thus, *B*={*x*}, and so $\left|\bar{B}\setminus Y\right|>1$ which is a contradiction.

**Corollary 5 .**

Let *X* be a Whyburn space. Then, the following statements are equivalent:

1. *X* is an Alexandroff space
2. *X* is a primal space

**Proof**

It is enough to see that an Alexandroff Whyburn space is a functionally Alexandroff space. Hence, by Theorem 8, |↓*x*| > 2 for any *x* ∈ *X*. Two cases arise as follows:

(a) If |↓*x*|=1, we take *f*(*x*)=*x*, and thus $\bar{\left\{x\right\}}=↓x=\left\{x\right\}=\left\{f^{n}\left(x\right),n\in \mathbb{N}\right\}$.
(b) If |↓*x*|=2, then ↓*x*={*x*, *y*}, and in this case, we take *f*(*x*)=*y*. So, $\bar{\left\{x\right\}}$  =  ↓*x*={*x*, *y*}={*x*, *f*(*x*)}={*f*^*n*^(*x*), *n* ∈ *ℕ*}. Indeed, *f*^2^(*x*) ∈ {*x*, *f*(*x*)} since *f*^2^(*x*)=*f*(*f*(*x*))=*f*(*y*) which is equivalent, by the construction of *f*.
  - *y*, if |↓*y*|=1, and thus *f*^2^(*x*)=*f*(*x*)
  - *z*, if |↓*y*|=2, and thus *z* ∈ ↓*y*\{*y*}⊆↓*x*\{*y*}={*x*}, and consequently, *f*^2^(*x*)=*x*

**Theorem 9 .**

Let *X* be an Alexandroff space. Then, the following statements are equivalent:

- *X* is a *QAP*-space
- ∀*x* ∈ *X* and ∀*y*, *z* ∈ ↓*x*, we have (↓*x*=↓*y*)∨(↓*x*=↓*z*)∨(↓*y*=↓*z*)

**Proof**

The first remark that *X* is Alexandroff if and only if *T*_0_(*X*) is Alexandroff and for any *x*, *y* ∈ *X*; we have *x* ≤ *y*⟺*μ*_*X*_(*x*) ≤ *μ*_*X*_(*y*).

- (*i*)⟹(*ii*). Let *x* ∈ *X* and *y*, *z* ∈ ↓*x*. Then, *μ*_*X*_(*z*), *μ*_*X*_(*y*) ∈ ↓*μ*_*X*_(*x*). Now, using Theorem 8, we get (*μ*_*X*_(*x*)=*μ*_*X*_(*y*))∨(*μ*_*X*_(*x*)=*μ*_*X*_(*z*))∨(*μ*_*X*_(*z*)=*μ*_*X*_(*y*)). Therefore, (↓*x*=↓*y*)∨(↓*x*=↓*z*)∨(↓*y*=↓*z*).
- (*ii*)⟹(*i*). Let *x* ∈ *X*. By Theorem 8, it is enough to see that |↓*μ*_*X*_(*x*)| ≤ 2. In this case, suppose that ↓*μ*_*X*_(*x*)⊇{*μ*_*X*_(*x*), *μ*_*X*_(*y*), *μ*_*X*_(*z*)}. Then, *y*, *z* ∈ ↓*x*, and thus, by hypothesis, the family {*μ*_*X*_(*x*), *μ*_*X*_(*y*), *μ*_*X*_(*z*)} is not pairwise distinct, as desired.

**Example 4 .**

**Figure 2:**
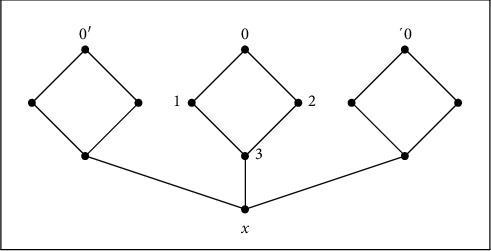
*QAP*-space.

**Figure 4:**
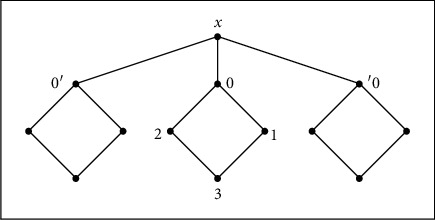
*X* is not a *QAP*-space.

**Figure 5:**
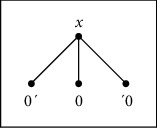
*T*_0_(*X*) is not a functionally Alexandroff space.

**Figure 6:**
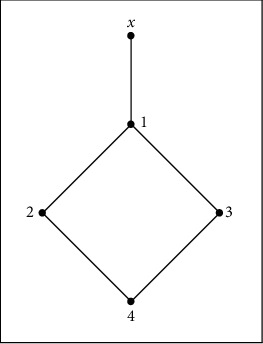
*X* is a *QAP*-space.

**Figure 7:**
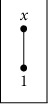
*T*_0_(*X*) is a functionally Alexandroff space.

(1) Figure 2shows the following: ↓*x*={*x*} and ↓0=↓1=↓2=↓3={0,1,2,3, *x*}. Let *a*, *b* ∈ ↓*t*, then two cases arise as follows:
  - If *t* ≠ *x*, then necessary ↓*a*=↓*t* or ↓*b*=↓*t*
  - If *t*=*x*, then *a*, *b* ∈ ↓*t* means that *a*=*b*=*t* and thus ↓*a*=↓*b*=↓*t*
(2)   Figure 4 shows the following:↓*x*=*X*, ↓0={0,1,2,3}, and ↓0′={0′, 1′, 2′, 3′}. Then, 1, 1′ ∈ ↓*x*, but {↓0, ↓0′, ↓*x*} is a family of pairwise distinct elements. Therefore, *X* is not a *QAP*-space.
   
*T*_0_(*X*) is defined by Figure 5which is not a functionally Alexandroff space.
(3) Figure 6 shows the following:
   
↓*x*=*X*, ↓4=↓1=↓2=↓3={1,2,3,4}, and thus, for any *a*, *b*, *c* ∈ *X*, we have (↓*a*=↓*b*)∨(↓*a*=↓*c*)∨(↓*b*=↓*c*). Therefore, *X* is a *QAP*-space.
   
*T*_0_(*X*) is defined by Figure 7 which is a functionally Alexandroff space.

**Theorem 10 .**

Let *X* be an Alexandroff space. Then, the following statements are equivalent:

- *X* is a *QWAP*-space
- *X* is a *QAP*-space

**Proof**

Using Proposition 1 and the fact that a topological space is Alexandroff if and only if its *T*_0_-reflection is Alexandroff. We get it immediately.

(7) $$\begin{array}{c} X\text{ is a QWAP}-\text{space}⟺T_{0}\left(X\right)\text{ is a WAP}-\text{space}\\ ⟺T_{0}\left(X\right)\text{ is a AP}-\text{space}\\ ⟺X\text{ is a QAP}-\text{space.} \end{array}$$

## Figures and Tables

**Figure 3:**
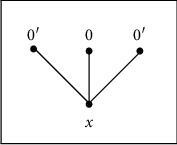
Functionally Alexandroff space.

## Data Availability

The data set can be accessed upon request.
